# A randomized controlled trial of a multimodality digital health intervention to aid the delivery of the modified ketogenic diet therapy for epilepsy: The Keto eCoach trial protocol

**DOI:** 10.1002/epi4.70345

**Published:** 2026-09-07

**Authors:** Neha Kaul, Lucy Vivash, Susanna Chen, Lillian King, Hannah Johns, Leonid Churilov, Zhibin Chen, John‐Paul Nicolo, Danielle Heaven, Ella Meredith, Ryan Ervine, Lisa Todd, Bernadette Zappa, Zanfina Ademi, Gopisankar Mohanannair Geethadevi, Michael Erlichster, Efstratios Skafidas, Terence J. O'Brien, Patrick Kwan

**Affiliations:** ^1^ Department of Neuroscience, School of Translational Medicine Monash University Melbourne Victoria Australia; ^2^ Department of Nutrition and Dietetics Alfred Heath Melbourne Victoria Australia; ^3^ Department of Neurology Alfred Health Melbourne Victoria Australia; ^4^ Department of Medicine, Royal Melbourne Hospital University of Melbourne Parkville Victoria Australia; ^5^ Department of Neurology Royal Melbourne Hospital Parkville Victoria Australia; ^6^ Australian Women with Epilepsy Ltd Bruswick East Victoria Australia; ^7^ Epilepsy Action Australia North Ryde New South Wales Australia; ^8^ Epilepsy Foundation Canterbury Victoria Australia; ^9^ Centre for Medicine use and Safety, Pharmacy and Pharmaceutical Sciences Monash University Melbourne Victoria Australia; ^10^ MX3 Pty, Ltd North Melbourne Victoria Australia; ^11^ Department of Electrical and Electronic Engineering University of Melbourne Parkville Victoria Australia; ^12^ Monash Institute of Medical Engineering Monash University Melbourne Victoria Australia

**Keywords:** dietary therapy, digital health, drug‐resistant epilepsy, ketogenic diet

## Abstract

**Objective:**

Dietary therapy is an effective treatment for drug‐resistant epilepsy; however, low adherence presents a significant barrier to use in the clinical setting. This study aims to evaluate a multimodality digital health intervention to overcome this barrier and improve outcomes in adults undertaking the modified ketogenic diet for epilepsy.

**Method:**

This is a two‐arm, randomized, controlled trial. A total of 136 participants will be randomized 1:1 to receive the modified ketogenic diet as per standard clinical care, or with the addition of the multimodality digital health intervention for 12 weeks. The intervention will include three components: (1) digital diaries, (2) a novel, at‐home, saliva‐based ketone testing device, and (3) dynamic dietitian support.

**Results:**

The primary treatment benefit will be determined using the epilepsy desirability of outcome rank, a composite outcome measure co‐designed with people living with drug‐resistant epilepsy. Secondary outcomes will include seizure frequency, treatment retention, adverse events, psychiatric symptoms, quality of life, medication use, cost effectiveness, and acceptability of the intervention.

**Significance:**

The results of this trial will provide evidence on the effectiveness of using this innovative digital health intervention approach to improve treatment outcomes of dietary therapy for drug‐resistant epilepsy.

**Ethics and Dissemination:**

This study has received approval from the Alfred Hospital Human Research Ethics Committee (Reference No.: 723/23). The results will be disseminated via peer‐reviewed publications and publicly available plain language summaries.

**Trial Registration Number:**

Australian New Zealand Clinical Trials Registry (ANZCTR, ACTRN12624000486527).

**Plain Language Summary:**

This trial aims to find out if a 12‐week digital health program can improve treatment outcomes of people living with epilepsy. We will invite 136 people commencing on the modified ketogenic diet to be randomly assigned to the digital health program or standard clinic care. The treatment outcome will be assessed combining seizure frequency, side effects, and quality of life.

**Key points**

- This is a randomized controlled trial evaluating a 12‐week multimodality digital health intervention program to support adults undertaking ketogenic diet therapy for drug‐resistant epilepsy.
- A total of 136 participants living with drug‐resistant epilepsy will be recruited.
- The treatment benefit will be assessed using a desirability of outcome rank, codesigned with consumers living with drug‐resistant epilepsy.
- If successful, this intervention could address the issue of poor adherence to ketogenic diet therapy that limits its effectiveness.
- The digital health delivery may also address geographical barriers to provision of ketogenic diet therapy.

## 1 INTRODUCTION

Anti‐seizure medications (ASMs) are the primary treatment for epilepsy. However, following multiple trials of medications, one‐third of patients continue to experience seizures and are considered to have drug‐resistant epilepsy.^1^, ^2^ The availability of newer ASMs has not changed the proportion of people living with drug‐resistant epilepsy.^3^

One of the established, safe, and effective non‐drug treatment options for drug‐resistant epilepsy is ketogenic diet therapies. Ketogenic diet therapy for epilepsy involves the manipulation of the proportions of the dietary macronutrients fat, protein, and carbohydrate. It can be administered through oral diet, tube‐feeding, intravenously, or in combination.^4^, ^5^ The most commonly used type of diet for adults with epilepsy is the modified ketogenic diet (MKD). The MKD is prescribed based on a macronutrient distribution of 75% fat, 20% protein, and 5% carbohydrate.^5^, ^6^

The effectiveness of ketogenic diet therapy for drug‐resistant epilepsy is supported by Level 1 evidence.^7^ In clinical trials, approximately 50% of patients using ketogenic diet therapy reported ≥50% reduction in seizure frequency and 10–15% became seizure‐free.^7^, ^8^ Other benefits include improved mood, cognition, and weight outcomes, in contrast to drugs and surgery, which often lead to neurocognitive complications or weight gain.^4^, ^5^ Ketogenic diet therapy is safe; side effects are generally mild and may include dyslipidaemia, unintentional weight loss, gastrointestinal intolerance, osteopenia, and kidney stones, which can be managed with proactive medical and dietetic monitoring. Recommendations for the use and monitoring of ketogenic diet therapies are endorsed by professional societies including the International League Against Epilepsy (ILAE) and patient advocacy groups.

Despite its demonstrated benefits, use of ketogenic diet therapy in the clinical setting is hampered by a number of factors. First, sustained adherence to the diet is low, particularly in adults, with 40%–60% of patients discontinuing treatment by 3–6 months.^7^, ^8^

Monitoring the efficacy and safety of the diet requires regular assessment of ketone levels, either by measuring β‐hydroxybutyrate (BHB) levels in blood or by aceto‐acetate levels in urine. Blood BHB concentrations of >2 mmol/L are associated with seizure reduction, whereas >5 mmol/L may result in metabolic acidosis.^9^ Although blood BHB monitoring is recognized as the “gold standard,” the procedure causes discomfort due to the finger‐prick and is expensive. Salivary BHB is a novel, accurate noninvasive alternative to blood BHB testing.^10^

Patients and caregivers have voiced that the current models of care do not provide optimal support. Typically, ketogenic diet therapy is commenced with an initial individual or group education session with the dietitian, advising of the diet prescription, implementation of the diet plan, food intake tracking, ketone monitoring and target levels, and monitoring and management of side effects. This is followed by outpatient follow‐up every 1–2 months. In between, patients and caregivers may or may not contact the dietitian on an ad‐hoc basis for further advice and troubleshooting.

Frequency of dietitian contact does not appear to influence treatment retention. Green et al.^11^ reported twice weekly telephone support for adults commencing on dietary therapy for epilepsy; 36% of participants ceased treatment within the first 12 weeks. The nutrition advice provided by the dietitian is often based on patient recall or paper‐based records. This makes it difficult for the clinician to systematically track and correlate dietary intake, ketone levels, side effects, and seizure frequency.

To overcome these barriers, this study will evaluate an innovative approach utilizing multimodality digital technologies to support the delivery of the MKD for drug‐resistant epilepsy compared to standard care.

### 1.1 Aims

The primary aim of this study is to investigate if 12 weeks of ketogenic diet therapy with multimodality enhanced technology (ET) support improves clinical outcomes in people with drug‐resistant epilepsy compared to standard‐of‐care ketogenic diet therapy alone. Improvement of clinical outcome will be defined using the epilepsy desirability of outcome rank (e‐DOOR) ordinal scale which has been co‐designed with people with drug‐resistant epilepsy.^12^

Secondary aims of the study relate to changes in seizure frequency, retention on ketogenic diet therapy, adverse events related to dietary therapy, quality of life, medication use, and direct and indirect healthcare costs. Acceptability of ketogenic diet therapy and the digital health intervention by participants will also be assessed.

## 2 METHODS

### 2.1 Ethics and trial registration

In February 2024, the trial received approval by the Alfred Hospital Human Research Ethics Committee (723/23). The trial was prospectively registered on the Australian New Zealand Clinical Trials Registry (ACTRN12624000486527). Prior to any study procedures, verbal and written consent will be obtained from the participant. If the participant is not capable of providing consent due to a cognitive impairment and/or intellectual disability, informed consent will be sought from the participant's legally authorized representative, such as a medical treatment decision maker (MTDM). Participants with cognitive impairment and/or intellectual disability may require assistance from a caregiver to complete the study requirements, such as implementing the MKD and completing the study diaries and questionnaires. In this situation, separate consent for the caregiver will be obtained. The MTDM and caregiver may or may not be the same individual.

### 2.2 Participant recruitment and eligibility criteria

To be eligible for inclusion in the trial, participants must fulfill all of the inclusion criteria and none of the exclusion criteria as outlined in Table 1.

**TABLE 1:** Participant inclusion and exclusion criteria.

To be eligible for inclusion in the trial, participants must fulfill all of the following inclusion criteria:
Male or female, aged ≥18 and <65 years. Diagnosis of drug‐resistant epilepsy according to ILAE^2^ definition, “failure of adequate trials of two tolerated and appropriately chosen and used anti‐seizure medication schedules (whether as monotherapies or in combination) to achieve sustained seizure freedom.” Must be under the care of a neurologist for their epilepsy treatment. Experiencing two or more seizures in the 4 weeks (past 28 days) prior to enrolment. Not currently on ketogenic diet therapy. No changes made to concomitant anti‐seizure medications or neuromodulation settings in the prior 4 weeks to enrolment. Access to a smartphone, tablet, and/or computer and internet access. Has a Medicare card. Is living in Australia. Proficient in speaking and reading English.
Participants will not be eligible to participate in the trial if they meet any of the following criteria:
Contraindications to ketogenic diet therapy for epilepsy, including fatty acid and/or amino acid oxidation or transport disorders. Current or previous diagnosis of an eating disorder. Pregnancy or breast‐feeding. Diagnosis of diabetes mellitus, serious hepatic or renal disease, or cancer. Current substance abuse disorder that may affect treatment adherence or response. Psychogenic nonepileptic (functional) seizures within the past 12 months. Participants will be excluded if baseline serum total cholesterol is >7.5 mmol/L, LDL cholesterol is >5.0 mmol/L, and/or triglycerides >5.6 mmol/L.

Abbreviation: ILAE, International League Against Epilepsy.

### 2.3 Study design

This is a two‐arm, randomized, controlled trial (Figure 1). The trial will be based at the Alfred Hospital in Melbourne, Australia. All study assessments can be completed at in‐person or by telemedicine visits ±7 days from the scheduled visit date. The number and timing of study visits will be the same in both treatment groups. A total of 136 participants will be recruited over 3 years.

**FIGURE 1:**
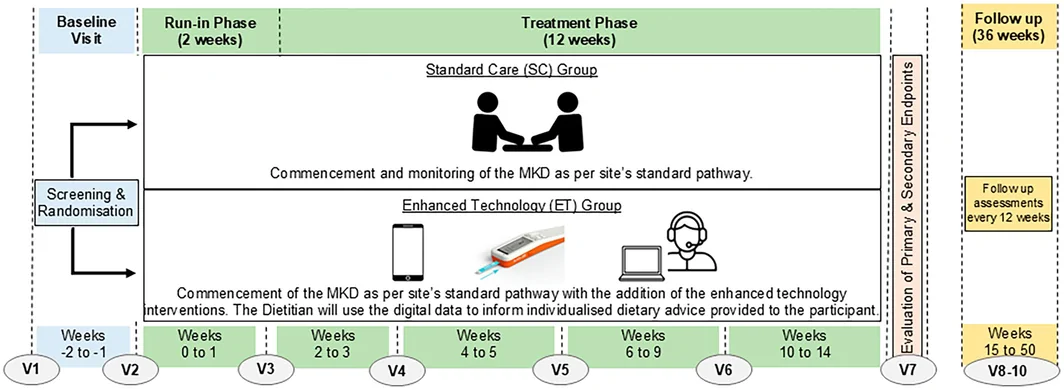
Study design and participant visits.

Eligible participants will be invited to take part in the trial and attend a baseline study visit (Week −2, Visit 1). After obtaining informed consent, demographic and clinical information will be collected. Participants will be provided with a blood test collection form. Eligible participants will be randomized to either the “enhanced technology” (interventional) group or standard care (control) group.

Participants will enter a prospective 2‐week baseline period (Week −2 to Week 0); during this time, they will maintain a daily seizure and food diary. They will be required to have their blood test during this time prior to the next study visit. The participants will receive their allocated ketone testing method via mail.

After 2 weeks, at Visit 2 (Week 0), participants will receive dietary education and begin to implement the dietary changes of the MKD over a 2‐week period (run‐in phase).

Following the run‐in phase, the participant will enter a 12‐week “treatment phase.” Study visits with the dietitian will be by telemedicine and occur at Week 2 (Visit 3), Week 4 (Visit 4), Week 6 (Visit 5), Week 10 (Visit 6), and Week 14 (Visit 7). Where possible, concomitant epilepsy treatment should remain stable; however, in emergency situations to maintain participant safety, medication changes occur as guided by their treating physician.

After completion of the 12‐week treatment phase, all participants who opt to continue on the MKD will be followed up in the Epilepsy Nutrition Clinic at Alfred Health. All participants will be requested to maintain a study diary recording seizure frequency, adverse events, and medications. Participants will be followed up by the dietitian via telemedicine every 12 weeks until week 50 to review their study diary and complete outcome assessments. All participants continuing on the MKD treatment will be provided with urine ketone testing strips and advised to test three times per week (Mondays, Thursdays, and Saturdays). All participants may contact the study dietitians at any time if issues arise.

### 2.4 Patient and public involvement statement

The study protocol was prepared in consultation and co‐designed with consumers. This process involved formation of a Consumer Advisory Committee which includes authors DH, EM, RE, LT, and BZ. The Consumer Advisory Committee members are people with lived experience of epilepsy and/or are representatives of the epilepsy advocacy agencies, Australian Women with Epilepsy, Epilepsy Foundation and Epilepsy Action Australia. The Consumer Advisory Committee has reviewed and approved the study protocol and study‐associated participant materials.

The primary outcome of the study is a novel consumer codesigned e‐DOOR specific for use in clinical trials in epilepsy. Details of the development of the e‐DOOR have been published elsewhere.^12^ In brief, the e‐DOOR is a composite outcome measure combining four levels of reduction in seizure frequency, five levels of adverse events, and three levels of quality of life, resulting in 60 combinations of potential outcomes, which are ranked from most desirable to least desirable (Table 2).

**TABLE 2:** Epilepsy desirability of outcome rank (e‐DOOR) (adapted from Vivash et al.^12^).

Outcome	Seizure frequency	Adverse events (AE)	Quality of life	Outcome rank
Levels	>90% reduction	No AE	Improvement	>90% reduction in seizure frequency, no AE and improvement in quality of life
	>50% reduction	Mild temporary AE	No change	2 >90% reduction in seizure frequency, no AE and no change in quality of life
	>25% reduction	Mild ongoing AE	Worsening	30. >90% reduction in seizure frequency, moderate ongoing AE, and worsening of quality of life
	<25% reduction	Moderate ongoing AE		59. <25% reduction in seizure frequency, severe ongoing AE, and no change in quality of life
		Severe ongoing AE		60. <25% reduction in seizure frequency, severe ongoing AE, and worsening in quality of life

Abbreviation: AE, Adverse events.

The results of the study will be presented at national and international scientific meetings and published in peer‐reviewed journals. In collaboration with the consumer advisory committee, the results will be publicized to the wider community.

### 2.5 Randomization and blinding

Participants will be randomized 1:1 using REDCap® to either the standard care arm (control) or ET arm (intervention group) for the treatment period. Randomization will be stratified for seizure frequency (greater or less than eight seizures per month during the baseline screening period), and requirement for an MTDM/caregiver. Participants will be randomly assigned to the treatment group as per a computer‐generated randomization schedule created by the study statistician (author HJ). The study dietitians (authors NK, SC, and LK) will be blinded to the randomization sequence but will be responsible for revealing the treatment group allocation to the participant. The participant and dietitian will be unblinded to the treatment allocation; the remaining study personnel will remain blinded.

### 2.6 Interventions

#### 2.6.1 Ketogenic diet therapy (all participants)

Following a comprehensive nutrition assessment by the dietitian, participants will be provided education on the MKD treatment. The MKD is prescribed based on a macronutrient distribution of 75%, 20%, and 5% of energy derived from fat, protein, and carbohydrate, respectively.^6^ The study dietitian will provide the initial education on implementation and monitoring of the dietary treatment. This includes meal planning, recipe modification, understanding food labels, and vitamin and mineral supplementation and review of adverse effects. Participants will use Easy Diet Diary® a smartphone application with verified nutrition composition data to plan and track dietary intake. Participants are provided with daily targets for total calories, and grams of fat, protein, and carbohydrate. Participants will be advised not to have dietetic interventions from another dietitian for the duration of time they are taking the MKD. The participant diet information sheet will be provided by email to the participant, and they will be offered a paper version to be mailed to them.

#### 2.6.2 Standard care (SC) group

As per current standard practice, the dietitian will use information gathered during the in‐person or telemedicine consultation to provide dietary advice for optimization of ketogenic diet therapy. Assessment of dietary intake will be based on food diary completed via Easy Diet Diary® smartphone application. Seizure frequency will be recorded on a paper‐based diary. Participants will be asked to measure their ketone levels every morning before having breakfast using urine ketone strips (Bayer Keto‐Diastix) and record the result in their paper‐based study diary.

#### 2.6.3 ET group

Participants in the ET group will receive the following additional ET interventions.

#### 2.6.4 ET intervention 1: Saliva ketone testing

Using the MX3 LAB Pro device (MX3 Diagnostics, Inc., Australia), participants will test their saliva every morning to monitor their fasting BHB levels with the target range between 32 and 678 mcmol/L (equivalent to 2.0–5.0 mmol/L capillary blood BHB). The results are available within 30 s and recorded in the participant's trial diary. Live and historical results will be available to the participant and study dietitian.

#### 2.6.5 ET intervention 2: Digital food and seizure diaries

Digital food and seizure diaries will be managed via REDCap.^13^, ^14^ Participants will log the time, date and type of seizure events, and medication use for the duration of the intervention using the digital diary. The participants will log and monitor their dietary intake of calories and grams of fat, protein, and carbohydrate in the digital diary as well as any adverse events.

#### 2.6.6 ET intervention 3: Dynamic dietitian support

The dietitian will have access to dietary intake information, seizure records, and saliva ketone levels prior to each study visit. This is in contrast to the standard care group, where this information is provided during the study visit, where participants provide a summary of their paper‐based seizure diaries and urine ketone testing results during the consultation. The dietitian will review the digital information prior to the study visit and provide individualized advice for the review consultations to further optimize dietary treatment. For example, if a participant had no change in seizure frequency and low saliva BHB concentration, dietary advice on further reduction in carbohydrate and increasing fat intake would be provided. Similarly, advice on management of side effects could be provided, such as increasing fiber and fluid intake to manage constipation. Early interventions to mitigate side effects may improve adherence and retention on dietary therapy.

### 2.7 Study assessments

All study outcome assessments will be conducted by an outcome assessor, blinded to the participant group allocation (Table 3).

**TABLE 3:** Schedule of study assessments.

Assessment/procedure	Screening	Run‐in phase	Start of treatment phase	Treatment phase			End of trial	Follow‐up phase		
	Visit 1 (Week 2)	Visit 2 (Week 0)	Visit 3 (Week 2)	Visit 4 (Week 4)	Visit 5 (Week 6)	Visit 6 (Week 10)	Visit 7 (Week 14)	Visit 8 (Week 26)	Visit 9 (Week 36)	Visit 10 (Week 50)
Informed consent	x									
Assess eligibility	x									
Confirm eligibility		x								
Demographic information	x									
Medical and epilepsy history	x									
Dietitian session	x	x	x	x	x	x	x	x	x	x
Food diary	x	x	x	x	x	x	x	x	x	x
Pathology	x						x	x	x	x
Plasma HCG	x									
Dispense study diaries Seizure record Food record Adverse events Medication use	x	x	x	x	x	x	x	x	x	x
Randomization	x									
Dispense urine Keto‐Diastix (standard group only)	x				x			x (all participants)	x (all participants)	
Dispense MX3 Lab Device and consumables (intervention group only)	x				x		x			
QOLIE‐31	x						x	x	x	x
EQ‐5D‐5L	x						x	x	x	x
CES (if applicable)	x						x	x	x	x
WPAI	x						x	x	x	x
Out of pocket costs and Informal care Questionnaires	x						x	x	x	x
Review of AEs/SAEs	x		x	x	x	x	x	x	x	x
Gastrointestinal symptoms questionnaire	x						x	x	x	x
Evaluation questionnaires							x			

Abbreviations: β‐hCG, beta‐human chorionic gonadotrophin; AE, adverse event; CES, Carer Experience Scale; EQ‐5D‐5L, EuroQol Five‐Dimensional Five‐Level; QOLIE‐31, Quality of Life in Epilepsy Inventory‐31; SAE, serious adverse event; WPAI, work productivity and activity impairment‐general health.

#### 2.7.1 Seizure diary

Participants will maintain a seizure diary for the duration of the trial. They will record the date, time, and type of seizures, either using a paper‐based diary during baseline screening and in the standard care group or a digital diary when on the ET intervention.

#### 2.7.2 Ketone level diary

Participants will maintain a ketone diary, commencing at Visit 2 (Week 0) until Visit 7 (Week 14), or the MKD is stopped, whichever event occurs first. Participants in the standard care group will record daily fasted urine ketone levels. Participants in the ET group will record daily fasted saliva ketone levels.

#### 2.7.3 Adverse events

Adverse events will be recorded in the participant's study diary. A questionnaire specifically focusing on gastrointestinal side effects will be provided at Visits 2, 7, 8, 9, and 10.

#### 2.7.4 Habitual diet

Regular dietary intake prior to commencement of the ketogenic diet will be assessed using a prospective 2‐week self‐reported food diary provided at Visit 1 (Week −2). Participants will be provided details on how to complete the food diary using Easy Diet Diary® app prior to Visit 2.

#### 2.7.5 Pathology safety monitoring

Pathology collection will occur at Visit 1 (Week −2), Visit 7 (Week 14), Visit 8 (Week 26), Visit 9 (Week 36), and Visit 10 (Week 50). Including blood urea and electrolytes, full blood examination, blood urea nitrogen, fasting lipid profile, fasting glucose, blood BHB, iron studies, zinc, selenium, ASM levels, carnitine profile, and beta‐hCG in women of childbearing potential. Blood collection can occur at the study site or at the participants' local pathology collection service.

#### 2.7.6 Psychiatric symptoms

The Neurological Disorders Depression Inventory in Epilepsy (NDDI‐E)^15^ and Brief Epilepsy Anxiety Survey Instrument (BrEASI)^16^ questionnaires will be completed at baseline (Week −2), at the completion of the treatment period (Week 14), and at each 12‐week follow‐up visit (Weeks 26, 36, and 50).

#### 2.7.7 Quality of life (QOL)

Questionnaires will be completed at baseline (Week 2), at the completion of the treatment (Week 14) at each 12‐week follow‐up visit (Weeks 26, 36, and 50). Health‐related QOL will be measured by both generic and epilepsy‐specific instruments. The EuroQol Five‐Dimensional Five‐Level (EQ‐5D‐5L)^17^ and Quality of Life in Epilepsy Inventory‐31 (QOLIE‐31)^18^ will be completed by the participant or proxy. Carer QOL will be measured by the Carer Experience Scale.^19^

#### 2.7.8 Acceptance of technology‐based intervention

An adapted questionnaire of the Theoretical Framework of Acceptability (TFA) questionnaire^20^ will be used to measure the acceptance of the intervention by participants, after the 12‐week intervention period (Visit 7). All participants will be asked to complete the acceptance of the ketogenic diet intervention questionnaire. Participants who were in the intervention group will also be asked to complete the acceptance of the digital health intervention questionnaire.

#### 2.7.9 Costs

Both direct healthcare costs and indirect costs will be collected. Direct healthcare costs will include epilepsy‐related clinic visits, visits to the general practitioner or other specialists, and emergency department presentations; use of chronic disease services; allied health services; and hospitalizations. Data linkage from the Medicare Benefits Scheme and Pharmaceutical Benefits Scheme will be utilized. The study dietitians will record the mode (e.g., in‐person, video call, phone or email), time (min) of encounters with trial participants of scheduled and unscheduled contact. Indirect healthcare costs will be collected using out‐of‐pocket costs and informal care questionnaires at Visits 2, 7, 8, 9, and 10.

Utilizing the quality‐of‐life measures (EQ‐5D‐5L for adults), a preference‐based scoring algorithm will be used to generate scores (0 = dead, 1 = full health scale) for economic evaluation. Indirect costs will be measured in terms of productivity loss due to seizure‐related absence from work or days of inactivity using the Work Productivity and Activity Impairment‐General Health^21^ at randomization (Week 0), at the completion of the treatment (Week 14), and at each 12‐week follow‐up visit (Weeks 26, 36, and 50).

### 2.8 Monitoring and data quality

An independent clinical trial monitor will conduct onsite monitoring. Onsite monitoring will ensure adherence to the study protocol and procedures from both a patient safety and data quality aspect. The frequency of the monitoring will be determined by the rate of participant recruitment, with the first monitoring visit occurring after the first three participants have completed the 12‐week treatment phase, and then after every 30 participants have completed the 12‐week treatment phase. A total of 27 participants (20% of all participants) will be audited for safety and data quality. A final monitoring visit will occur after the final participant has completed their last study assessment and prior to data lock. The clinical trials monitor will provide a report at the end of each monitoring visit. These reports will be reviewed at the quarterly steering committee meetings.

## 3 ENDPOINTS AND STATISTICAL ANALYSIS OVERVIEW

### 3.1 Power and sample size

Power analysis for the win ratio outcome requires the alternative hypothesis about the combination of proportions of all one‐to‐one comparisons to a random participant in the standard care arm, where a random participant receiving the ET intervention will have a better, worse, or same epilepsy‐DOOR scale outcome, respectively (proportions of wins, losses, and ties). We hypothesize that the ET intervention will result in a distribution of win/draw/loss proportions of 0.6:0.1:0.3 that corresponds to the Win ratio = 0.6/0.3 = 2 accounting for potential ties.

Recruiting 108 participants would provide a power of 0.8 to detect the hypothesized treatment effect, testing against the null hypothesis of no treatment effect (Win ratio = 1), assuming a two‐sided alpha level of 0.0533. The final sample size is set at 136 participants distributed between intervention and control, accounting for 20% potential losses due to screening failures and participant withdrawal from the study.

### 3.2 Primary end point

The odds that a random participant who received ketogenic diet therapy with the addition of the ET intervention will have a more desirable epilepsy‐DOOR scale outcome than a random participant who received ketogenic diet therapy as per standard care at 12 weeks from the start of the intervention.

### 3.3 Secondary end points

1. *Seizures*: Proportion of participants with ≥50% and 100% (seizure‐free) reduction in seizure frequency per 28 days after 12 weeks dietary therapy will be compared between groups.
2. *Retention on dietary therapy*: Proportion of participants remaining on the MKD after 12 weeks of treatment will be compared between groups.
3. *Adverse events related to dietary therapy*: The total number and severity of adverse events will be compared between groups after 12 weeks of treatment.
4. *Psychiatric symptoms and QOL*: The proportion of participants experiencing a clinically meaningful improvement in psychiatric symptoms and QOL questionnaire scores will be compared between groups after 12 weeks of treatment.
5. *Regular and rescue medication use*: Change in the number of regular medications and instances of rescue medication use will be compared between groups after 12 weeks of treatment.
6. *Cost‐effectiveness evaluation*: Direct and indirect healthcare costs and loss of productivity will be compared between groups after 12 weeks of treatment.
7. *Acceptance of technology‐assisted healthcare delivery*: The Theoretical Framework of Acceptability (TFA) questionnaire^20^ will be used to assess the acceptability of ketogenic diet therapy and the ET intervention by participants.

### 3.4 Analysis

Intercurrent events causing early discontinuation will be handled under the International Council for Harmornisation E9 (R1)^22^ Estimand Framework, with appropriate intercurrent event strategies used for each type of intercurrent event. Missing data for the primary analysis, and all components of the Epilepsy‐DOOR, will be handled using multiple imputation. Full details of the statistical analysis, including intercurrent event definitions and strategies for handling them as well as missing data handling, will be prespecified in the statistical analysis plan prior to database lock.

## 4 LIMITATIONS

We acknowledge the potential limitations in the study design, some of which are unavoidable due to the nature of dietary interventions. First, the lack of participant and dietitian blinding to the treatment allocation. This has been partially mitigated by the outcome assessor and statisticians remaining blinded. We acknowledge that the use of a digital health intervention may introduce selection bias of participants. The open‐label study design and use of patient‐reported outcome measures may introduce reporting bias; to minimize this, we have used validated questionnaires. Finally, the multiple components of the digital health intervention make it difficult to disentangle which specific element of the intervention is responsible for any observed effect.

## 5 SIGNIFICANCE

This randomized controlled trial will generate level 1 evidence regarding the effectiveness of using this innovative digital health intervention approach in supporting ketogenic diet therapy for drug‐resistant epilepsy management. Our trial design allows for the robust evaluation of the ET model of ketogenic diet therapy delivery compared to current standard care. If the ET model is found to be effective, it will overcome one of the major barriers that limit the effectiveness of dietary therapy for epilepsy. Reducing seizure frequency in patients with drug‐resistant epilepsy will have a significant impact on the individual by reducing the risk of prolonged seizures requiring hospitalization, injuries, exposure to medication and their side effects, and death and reduce costs across the healthcare system. If demonstrated to be effective, this approach may be utilized in other conditions with high response to diet‐based treatments, such as diabetes, obesity, inborn errors of metabolism, or malnutrition.

## AUTHOR CONTRIBUTIONS

N.K. drafted the first manuscript. N.K., T.O.B., and P.K. conceptualized the study. N.K., L.V., L.K., S.C., H.J., L.C., Z.C., J.‐P.N., D.H., E.M., R.E., L.T., L.W., Z.A., G.M.G., M.E., E.S., T.O.B., and P.K. contributed to the study design. T.O.B. and P.K. provided supervision. All authors critically revised the manuscript and approved the final version.

## FUNDING INFORMATION

The trial is funded by a Medical Research Future Fund grant provided by the National Health and Medical Research Council (MRF2024901).

## CONFLICT OF INTEREST STATEMENT

MX3 Diagnostics manufactures the MX3 LAB the device used in this study. ES is a member of the MX3 Diagnostics Board of Directors. M.E. is an employee of MX3 Diagnostics. E.S. and M.E. have partial ownership of MX3 Diagnostics. E.S. and M.E. are authors of patents related to MX3 products. The other authors declare no conflicts of interest. We confirm that we have read the Journal‘s position on issues involved in ethical publication and affirm that this report is consistent with those guidelines.

## Data Availability

Data sharing not applicable to this article as no datasets were generated or analysed during the current study.
